# Synthesis and Unique Behaviors of High-Purity HEA Nanoparticles Using Femtosecond Laser Ablation

**DOI:** 10.3390/nano14060554

**Published:** 2024-03-21

**Authors:** David Fieser, Yucheng Lan, Antonino Gulino, Giuseppe Compagnini, Doug Aaron, Matthew Mench, Denzel Bridges, Hugh Shortt, Peter Liaw, Anming Hu

**Affiliations:** 1 Department of Mechanical, Aerospace, Biomedical Engineering, The University of Tennessee, Knoxville, 1512 Middle Drive, Knoxville, TN 37996, USA; davjfies@vols.utk.edu (D.F.);; 2 Department of Physics and Engineering, Morgan State University, 1700 East Cold Spring Lane, Baltimore, MD 21251, USA; 3 Chemistry Department, University of Catania, Viale A. Doria 6, 95125 Catania, Italy; 4 Department of Materials Science and Engineering, The University of Tennessee, Knoxville, 1512 Middle Drive, Knoxville, TN 37996, USA

**Keywords:** femtosecond laser, laser ablation, high-entropy alloy, nanoparticle synthesis, electrocatalysis, scalable synthesis, oxygen reduction reaction

## Abstract

High-entropy alloys (HEAs) are a class of metal alloys consisting of four or more molar equal or near-equal elements. HEA nanomaterials have garnered significant interest due to their wide range of applications, such as electrocatalysis, welding, and brazing. Their unique multi-principle high-entropy effect allows for the tailoring of the alloy composition to facilitate specific electrochemical reactions. This study focuses on the synthesis of high-purity HEA nanoparticles using the method of femtosecond laser ablation synthesis in liquid. The use of ultrashort energy pulses in femtosecond lasers enables uniform ablation of materials at significantly lower power levels compared to longer pulse or continuous pulse lasers. We investigate how various femtosecond laser parameters affect the morphology, phase, and other characteristics of the synthesized nanoparticles. An innovative aspect of our solution is its ability to rapidly generate multi-component nanoparticles with a high fidelity as the input multi-component target material at a significant yielding rate. Our research thus focuses on a novel synthesis of high-entropy alloying CuCoMn_1.75_NiFe_0.25_ nanoparticles. We explore the characterization and unique properties of the nanoparticles and consider their electrocatalytic applications, including high power density aluminum air batteries, as well as their efficacy in the oxygen reduction reaction (ORR). Additionally, we report a unique nanowire fabrication phenomenon achieved through nanojoining. The findings from this study shed light on the potential of femtosecond laser ablation synthesis in liquid (FLASiL) as a promising technique for producing high-purity HEA nanoparticles.

## 1. Introduction

In nanomaterial science, developing methods for the rapid and reliable synthesis of nanoparticles, especially from complex materials like HEAs, is essential [1,2]. HEAs, with their composition of four or more principal elements in nearly equal molar ratios, have significantly altered the traditional approach to alloy design. These alloys are distinguished by their diverse multi-element composition, leading to an array of desirable physicochemical and mechanical properties [3,4]. Their versatile application potential spans a range of industries, including high-temperature materials, anti-corrosion, electrocatalysis, advanced welding, and brazing [5,6,7,8,9]. The real power of HEAs lies in their high-entropy effect, which promotes the formation of simple solid phases over a more intermetallic composition, thereby granting these alloys remarkable characteristics. In our study, we selected an HEA composed of CuCoMn_1.75_NiFe_0.25_ due to its potential catalytic properties stemming from its composition, its effectiveness in brazing and welding applications, and the high probability of being single phase [10,11,12,13,14,15]. Also, this composite includes Mn, an element with the potential to catalyze ORR [16,17]. Unlocking the full potential of HEAs necessitates sophisticated synthesis techniques that preserve their complex composition at the nanoscale [18,19]. The essence of HEAs lies in their ‘high-entropy effect’, which stabilizes simple solid solutions, leading to versatile microstructures. This is complemented by three additional effects: the sluggish diffusion effect, which enhances high-temperature stability; the severe lattice distortion effect, contributing to their mechanical strength; and the cocktail effect, which allows for the fine-tuning of properties by altering its elemental makeup. These four core effects not only underscore HEAs’ adaptability across various applications, but also highlight the need for advanced synthesis techniques [20]. Such methods must preserve the complex, multi-element composition of HEAs at the nanoscale, a crucial step for leveraging their full potential in cutting-edge material applications [21,22].

Researchers have developed a range of techniques to synthesize high-entropy alloy nanomaterials, addressing the increasing demand for efficient, single-phase HEA nanomaterials. Developed methods include carbothermal shock (CTS) [23], mechanical alloying [24,25], plasma-spark sintering [24], microwave heating [26], wet chemistry [27], and laser ablation synthesis [28]. Each method offers distinct advantages in process control, scalability, processing time, yield, and property customization, but also includes limitations like potential contamination, excessive energy requirements, and post-processing needs [2]. Using the CTS method, exposure of the carbon substrate can lead to unwanted reactions, which poses challenges for applications such as hydrogen evolution, CO_2_ reduction, and N_2_ reduction reactions, where high nanoparticle surface coverage is essential [29]. Mechanical alloying can be time-intensive and has a risk of contamination [12,24]. The effectiveness of microwave heating varies with material properties; wet chemistry faces challenges in nanoparticle size control and environmental safety [30,31]. Laser ablation synthesis, known for its precision and minimal contamination risk, has high energy requirements and is prone to producing inconsistent particle sizes. The above challenges need to be addressed to make HEA nanomaterial synthesis more viable for widespread industrial applications [32].

To address these challenges, recent research has shifted toward exploring innovative methods that mitigate such limitations. This focus is driven by the need for more precise, scalable, and environmentally friendly approaches in HEA nanomaterial synthesis. The following studies showcase various advancements and methodologies in the field of laser-based nanoparticle synthesis. Redka et al. [33] focus on nanoparticle synthesis through single-pulse, sub-picosecond laser ablation of an HEA (CrMnFeCoNi) and stainless steel AISI304. Their study provides detailed insights into the synthesis process, specifically highlighting the energy-specific ablation volume and the influence of material properties on ablation efficiency and crater morphology. The study shows that the CrMnFeCoNi alloy has a slightly lower ablation threshold than AISI304, resulting in larger crater diameters, indicating its efficient processability with ultrashort-pulse lasers. Waag et al. [28] present a detailed study on the synthesis of colloidal HEA nanoparticles using a kinetically controlled laser method. They focus on synthesizing the CoCrFeMnNi HEA nanoparticles with equimolar stoichiometry and diameters below 5 nm. The method involves liquid-phase ultrashort-pulsed laser ablation of consolidated and heat-treated micropowders of constituent metals. Their study demonstrates the processing scalability and potential for producing nanoparticles with unique redox behaviors, potentially useful in heterogeneous catalysis for oxygen evolution reactions (OERs). This approach marks a significant advancement in the controlled synthesis of HEA nanoparticles. Rawat et al. [34] explore the formation of Cu-Ni-enriched phases in nonequiatomic AlSiCrMnFeNiCu HEA nanoparticles through laser processing. They examine the structural and compositional changes induced by the laser treatment, emphasizing the formation of Cu-Ni nanoprecipitates. Their study is significant for its insights into the manipulation of nanoparticle composition and structure using laser techniques, with potential applications in various fields due to the unique properties of HEAs. Jahangiri et al. [28] focus on the synthesis of HfNbTaTiZr refractory high-entropy alloy nanoparticles using femtosecond laser-mediated preparation. They study the effects of different solvents and laser fluence on the nanoparticles’ properties, with an emphasis on applications in photothermal therapy. Their research demonstrates the use of synthesis methods to produce nanoparticles with varying structural and compositional characteristics, influenced by the choice of solvent and laser parameters.

Femtosecond laser technology presents a potential solution to these challenges [27,28]. Femtosecond lasers, characterized by ultrashort pulse durations, require significantly lower average power compared to conventional continuous wave laser or pulsed lasers with a long pulse width [35,36]. This advancement not only reduces energy consumption but also enhances the control over particle size and distribution, paving the way for more efficient and precise HEA nanoparticle synthesis as well as for many other fields of laser manufacturing and processing [37,38].

In our research, we proposed and tested a novel femtosecond laser ablation synthesis method in liquid, specifically tailored for the efficient synthesis of high-entropy alloy nanoparticles. This approach utilized a femtosecond laser to precisely ablate an HEA alloy within a liquid environment. We first concentrated on fundamentally understanding the ablation process and optimizing it by experimenting with various liquid media, aiming to identify the most effective conditions for nanoparticle production. A key aspect of our solution was its ability to rapidly generate nanoparticles with a high fidelity as the input target material at a significant rate, thereby achieving a production speed of more than 200 mg per minute. Moreover, we designed this process to be continuously operable, aiming to ensure a steady and efficient generation of high-quality single-phase nanoparticles. Our method has also reported the unique fabrication of nanoparticle chains fabricated through laser-induced nanojoining [39,40]. This method, now proven successful, has the potential to revolutionize the production of HEA nanoparticles, offering a scalable and consistent approach for industrial applications.

## 2. Materials and Methods

### 2.1. Material Synthesis

#### 2.1.1. Bulk Material of HEA

A cylindrical CuCoMn_1.75_NiFe_0.25_ high-entropy alloy with a diameter of 9 mm and length of 40 mm was produced using a 10 g titanium-gettered arc melter (MRF—Materials Research Furnaces Inc., Allenstown, NH, USA) at 600 amperes. This process employed a quarter-inch diameter lanthanated tungsten tip to ensure effective mixing. The arc melter’s vacuum chamber underwent oxygen purging for one hour, reaching a vacuum pressure of 50 mTorr with the aid of a roughing pump. It was then backfilled to 0.5 atmosphere using high-purity argon (99.999% by volume) with a 5 s fill time before each melting cycle, which lasted a minimum of 20 s in a copper mold. The process included 5 min water cooling intervals after each of the seven flips to achieve a homogenous mix. The HEA’s constituent elements, namely, Co (99.95% metals basis), Cu (99.9% metals basis), Mn (99.95% metals basis), Ni (99.98% metals basis), and Fe (99.95% metals basis), were measured with 1 mg accuracy.

#### 2.1.2. HEA Nanoparticle Synthesis through Laser Ablation

**Figure 1:**
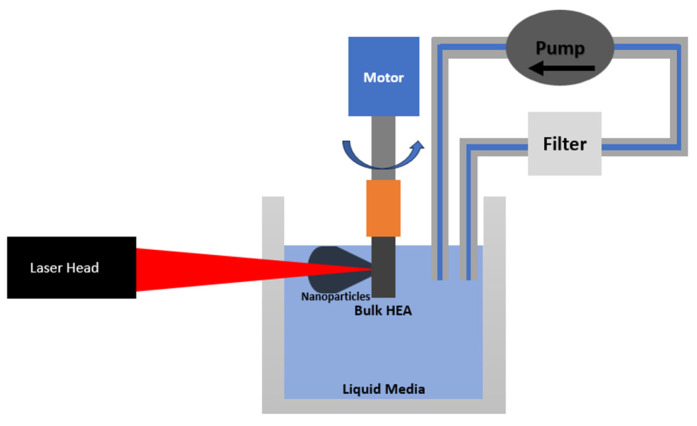
Schematic of the HEA ablation system.

In our synthesis method for HEA nanoparticles, a femtosecond laser was directed onto the surface of a cylindrical bulk HEA, which was fully immersed in a liquid medium. To ensure consistent ablation, the sample was rotated at a tunable speed up to nearly 9000 rotations per minute. As a result, the nanoparticles produced were immediately submerged in the surrounding liquid medium. This medium, along with the nanoparticles, was then extracted and collected using Whatman 1001-0155 cellulose filter paper, which was sourced from McMaster-Carr, Santa Fe Springs, CA, USA. The filter paper, characterized by its 180 μm thickness, 55 mm diameter, and notably large 11 μm pore size, proved effective in capturing the nanoparticles despite its relatively large pores. An additional parameter influencing ablation performance was the mixing of the ablated material within the liquid medium over time. As nanoparticles accumulated, transparency decreased—attenuating laser propagation and slowing down ablation. Within this work, we utilized continuous filtration to remove nanoparticles, improving optical penetration. The device is detailed in Figure 1.

Laser ablation in this study was conducted using a unique femtosecond laser system (100 W 500 fs Amplitude Tangor 100^®^ Amplitude Laser Inc., Milpitas, CA, USA) under a range of average power levels and frequencies, with a consistent pulse width of 500 fs and a laser focal diameter fixed at 50 μm. The average laser power settings varied from 0.2 W to 50 W, while the frequency range was maintained between 50 kHz and 100 kHz.

### 2.2. Microstructure, Phase, and Electrochemical Characterization

Scanning electron microscopy (SEM) was conducted with a ThermoFisher Scientific Helios 5 Dual-Beam system (Thermo Fisher Scientific, Waltham, MA, USA). Transmission electron microscopy (TEM) was performed with a Zeiss Libra200 (Zeiss Group, Oberkochen, Germany). The phase compositions of the welds were analyzed by X-ray diffraction using a Malvern Panalytical Empyrean diffractometer with Cu-Kα radiation (Malvern Panalytical Ltd., Malvern, United Kingdom). Particle size analysis was performed with ImageJ software V 1.8.0.

The catalytic performance of HEA nanoparticles was characterized using a rotating disk electrode (RDE) with a diameter of 5 mm made of glassy carbon. Catalyst ink was made by mixing HEA nanoparticles and Ketjen Black (KB) in ethanol, maintaining a weight ratio of 2:1. Prior to the dispersion of the nanoparticles, 10 µL of Nafion solution was incorporated into the ethanol solvent to enhance the stability of the catalyst layer. A total of 150 µg of the catalyst, with a concentration of 3 µg/µL, was applied to the electrode in increments of 10 µL, applied dropwise onto the desired surface in five separate drops for uniform coverage. After application, the electrode was left to dry for two hours. Electrochemical tests were conducted for both the ORR and the oxygen evolution reaction (OER), as well as for the hydrogen evolution reaction (HER). These tests were performed at a consistent rotational speed of 1600 RPM. ORR tests took place in an oxygen-saturated environment, using a 0.1 M KOH electrolyte. In contrast, OER and HER tests required the electrolyte to be degassed with nitrogen, with OER tests also using 0.1 M KOH, while HER tests were conducted in 0.5 M KHCO_3_. We employed linear sweep voltammetry (LSV), utilizing a Biologic-SP200 potentiostat, to evaluate the electrochemical performance of our system. Tests were performed over a wide voltage range to facilitate separate chemical reactions. ORR tests were conducted at 1.5 volts, down to 0 volts, HER testing was conducted starting at 0.25 W and swept down to −1 V, and OER started at 1 V and swept to 2 V. To accurately reference the electrochemical potentials during our experiments, a Hg/HgO reference electrode was employed, set at a potential of +1.44 V, according to the Nernst equation.

Additional electrochemical characterizations were performed using an in-house constructed aluminum–air (Al–air) battery flow battery system, measured with battery testing apparatus [16]. This system featured an anode made from an Al 7075 anode and a cathode comprised of carbon fiber paper (CFP, model TGP-H-060 Fuel Cell Earth LLC, Woburn, MA, USA) onto which our synthesized HEA nanoparticle catalyst ink was deposited. The electrolyte used was an alkaline aqueous solution of 6 M KOH. Prior to assembly, the carbon fiber papers serving as the cathode were meticulously cleaned using ultrasonic agitation in ethanol, followed by drying. The catalyst ink layer was prepared by creating an ink composed of HEA nanoparticles, KB, and carbon Super-P at a weight ratio of 3:3:1. This mixture was then dispersed in pure ethanol to achieve a concentration of 1 mg/mL. A total of 1 mL of the ink was carefully applied to the CFP in a controlled, dropwise manner. For the anode, a commercially available Al 7075 plate measuring 2 cm by 2 cm by 6 mm was utilized. This configuration of anode and cathode, coupled with our unique catalyst composition, was integral to evaluating the electrochemical performance of the HEA nanoparticles in our Al–air battery system. To evaluate the electrochemical performance of our system, linear sweep voltammetry was employed, utilizing a Biologic-SP200 potentiostat (BioLogic USA, Knoxville, TN, USA). The tests began with an initial voltage close to the open-circuit voltage, approximately 1.5 V, and progressed down to 0 V. We performed these measurements at a consistent scan rate of 10 mV/s. This methodical approach allowed for a detailed analysis of the electrochemical characteristics of our setup under controlled conditions.

## 3. Results and Discussion

### 3.1. Morphology, Phase, and Composition

**Figure 2:**
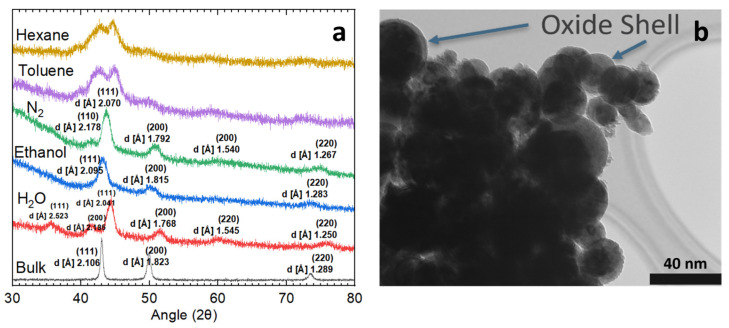
(**a**) XRD analysis of femtosecond-ablated high-entropy alloy nanoparticles in diverse media, including bulk composition. (**b**) High-entropy alloy nanoparticles ablated in water exhibiting a core–shell structure.

The nanoparticles obtained from the experiments were subjected to X-ray diffraction analysis along with the original face-centered cubic (FCC) bulk HEA [a = 3.6476 Å], the results of which are depicted in Figure 2a. The initial ablation experiments were conducted in deionized water. XRD analysis reveals a multi-phase composition identifiable as a metal phase [FCC, a = 3.5360 Å] and a metal oxide phase [FCC, a = 4.3700 Å]. Further structural insights were gained through TEM, shown in Figure 2b, which uncovered a core–shell configuration of the nanoparticles. Accordingly, this core–shell structure is characterized by a solid metal core surrounded by a metal oxide shell, with the indexed peaks presented in Figure 2a. Laser-induced periodic surface structures with characteristic ripples form on the HEA surface during ablation (see Supplementary Figure S1). Morphological changes at the target–liquid interface can influence plasma ejection dynamics. In turn, this provides potential routes to intentionally tailor nanoparticle size distributions or composition. However, the underlying physics of the relationship between the surface ripple and the morphology of the ablated nanoparticles is worthing further investigation [41,42,43].

### 3.2. Nanoparticle Composition

**Figure 3:**
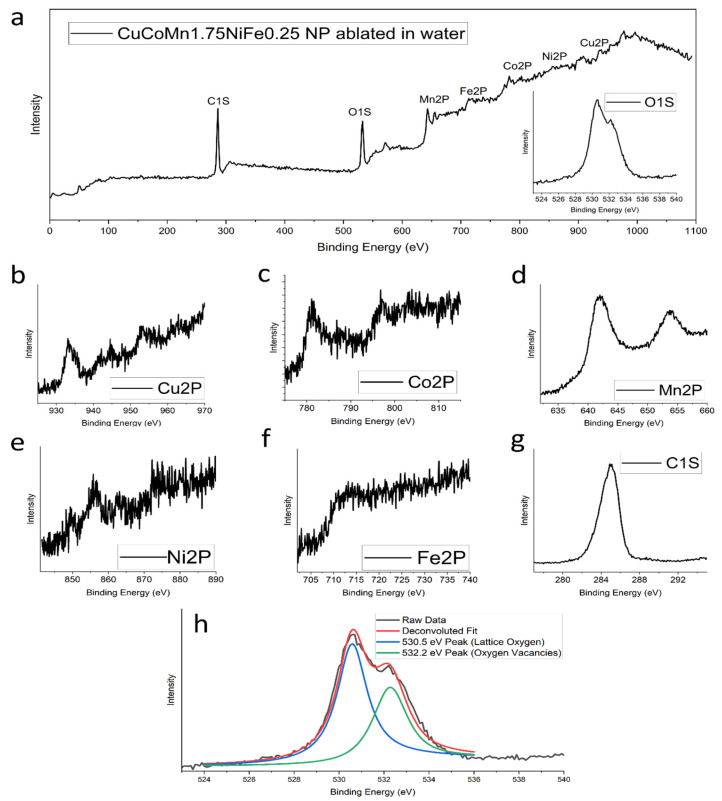
XPS analysis of CuCoMn_1.75_NiFe_0.25_ nanoparticles ablated in water. (**a**) Survey scan, with O-1s subplot. (**b**) Cu-2p. (**c**) Co-2p. (**d**) Mn-2p. (**e**) Ni-2p. (**f**) Fe-2p. (**g**) C-1s. (**h**) O-1s deconvolution.

**Figure 4:**
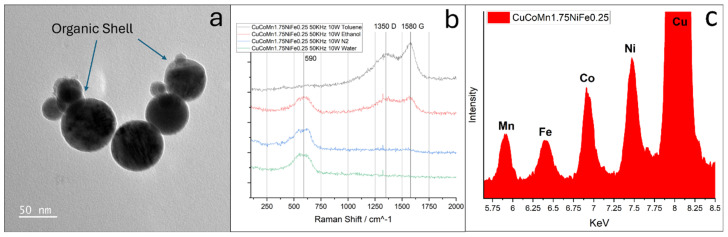
(**a**) High-entropy alloy (HEA) nanoparticles ablated in ethanol with an organic shell encasing a metallic core. (**b**) Raman shift spectra of HEA nanoparticles ablated in different media, displaying carbon D and G bands. (**c**) EDS of ceramic shell nanoparticles showcasing all five elements.

To comprehensively examine both the surface states and internal composition of the nanoparticles, X-ray photoelectron spectroscopy (XPS) analysis was conducted. The findings from this analysis are presented in Figure 3. Notably, the XPS peaks corresponding to the metals are evident in parts (b–f) of Figure 3. Analysis of these curves reveals the normalized ratio of the metals present in the nanoparticles as 1:0.89:1.97:0.93:0.37. This observed ratio also aligns with the intended stoichiometric composition of CuCoMnNiFe, which is 1:1:1.75:1:0.25, indicating a high degree of accuracy in nanoparticle synthesis. The oxygen orbital scans can be identified in Figure 3h. Two deconvoluted peaks are observed. The presence of two distinct peaks in the XPS scan provides valuable insights into the chemical structure. The first peak, observed at around 530.5 eV, is indicative of the standard bonding environment of oxygen in the alloy oxide. This peak represents the oxygen atoms that are fully integrated into the lattice structure of the oxide phase, reflecting a stable, typical oxide composition. The second peak, appearing at 532.2 eV, is particularly interesting as it signifies the presence of oxygen vacancies within the oxide structure. In the physical sense, oxygen vacancies are essentially spots in the oxide lattice where oxygen atoms would be expected but are absent. The ratio of the intensities of these two peaks (at 530.5 eV and 532.2 eV) can be used as a quantitative measure of the oxygen vacancy ratio in the oxide phase [44]. A higher intensity of the peak at 532.2 eV, relative to the peak at 530.5 eV, suggests a greater proportion of oxygen vacancies. These vacancies can significantly change the material’s properties, such as its ability to conduct electricity, its magnetic behavior, and its catalytic activity in speeding up chemical reactions [45,46,47,48]. In this case, the ratio of oxygen vacancies to lattice oxygen is 0.78, indicating a substantial presence of oxygen vacancies in the material. The XPS results corroborate the presence of all constituent elements in the bulk high-entropy alloy. As depicted in Figure 3, the emergence of peaks corresponding to each element in the bulk material validates the successful synthesis of proper HEA nanoparticles. Peaks for copper, cobalt, manganese, nickel, and iron are visible. Since our composition has high amounts of manganese and low amounts of iron, the evidence of high fidelity is demonstrated by the strength and clarity of the peaks associated with manganese, and the weak peaks associated with iron. EDS measurements on TEM do not identify remarkable compositional differences among these nanoparticles. The presence of carbon can be attributed to possible surface contamination during sample transportation. Raman spectroscopy analyses were conducted on this nanoparticle composition immediately after synthesis, with the results presented in Figure 4b. These findings reveal an absence of significant carbon signals, reinforcing the assertion that the carbon detected in the XPS analysis likely originates from later surface adsorption rather than being an integral component of the nanoparticles.

The investigation into water media-ablated nanoparticles yielded intriguing and potentially beneficial outcomes, yet the resulting nanoparticles were not purely metallic. Exploration was extended to other liquid media, encompassing liquid nitrogen, ethanol, and various hydrocarbon liquids. Although initially a promising concept, especially due to its oxygen-free nature, the nanoparticles produced in liquid nitrogen underwent phase separation. This separation manifested as distinct pure metal and nitride phases [FCC, a = 4.7241 Å]. This phenomenon is clear in Figure 2a, providing confirmation of the impurity phase. When samples were ablated in toluene and hexane, the resulting nanoparticles exhibited significant carbide content, along with the presence of unbound organic materials interspersed among them. These compositions are also represented in Figure 2a. A distinct phase separation is observable in these lines, further highlighted by the emergence of a clearly defined amorphous region. This latter feature is particularly evident in the lower angle region, spanning from 30° to 40°, where there is increased scattering. Samples ablated in ethanol had the most promising results, with a clear single-phase metal composition [FCC, a = 3.630 Å]. An amorphous region is still visible in the blue line in Figure 2a. This trend was theorized to be an organic shell present around a metal core. The investigation of the nanoparticle morphology was conducted using TEM, as depicted in Figure 4a. This image reveals a distinct thin shell encircling the nanoparticle’s core. To look deeper into these findings, Raman spectroscopy was employed on nanoparticles extracted from various media. The spectrum shown in Figure 4b for ethanol corresponds to unwashed nanoparticles ablated in ethanol, where a pronounced presence of carbon D and G bands is evident. The detection of the D and G bands in the Raman spectrum is a compelling indication of the presence of organic material. The D band, which typically appears around 1350 cm^−1^, is characteristic of disordered or amorphous carbon structures and serves as an indicator of organic compounds that have not attained a fully crystalline graphite structure [49,50]. Conversely, the G band, located near 1580 cm^−1^, represents the graphitic or more ordered carbon structures. This band is associated with the vibrations of sp2-bonded carbon atoms in a two-dimensional hexagonal lattice, which is a hallmark of graphitic carbon, commonly present in organic materials [49,50]. This result is different to those obtained from femtosecond laser ablation of Fe nanoparticles, where a hexagonal diamond shell is reported [51]. The difference may be attributed to various laser pulse widths and pulse energies since the carbon phase is dependent on the Coulomb explosion of organic molecules [50]. The concurrent presence of both the D and G bands suggests a composite carbonaceous nature of the nanoparticles, encompassing both ordered and disordered carbon forms.

In conjunction with this study, energy-dispersive X-ray spectroscopy (EDS) was also employed on TEM as a complementary technique to verify the presence of all anticipated elements within the sample, as illustrated in Figure 4c. This figure specifically emphasizes the Kα orbital transitions of the elements under investigation. The EDS experiment shows the clear presence of copper, cobalt, manganese, nickel, and iron. The peak associated with copper stands out because it is strengthened by the presence of copper in the TEM grids. The preliminary EDS spectrum, as highlighted, reveals distinct peaks corresponding to these Kα transitions, providing clear evidence for the existence of each element within the sample. The quantitative analysis is still under way.

**Figure 5:**
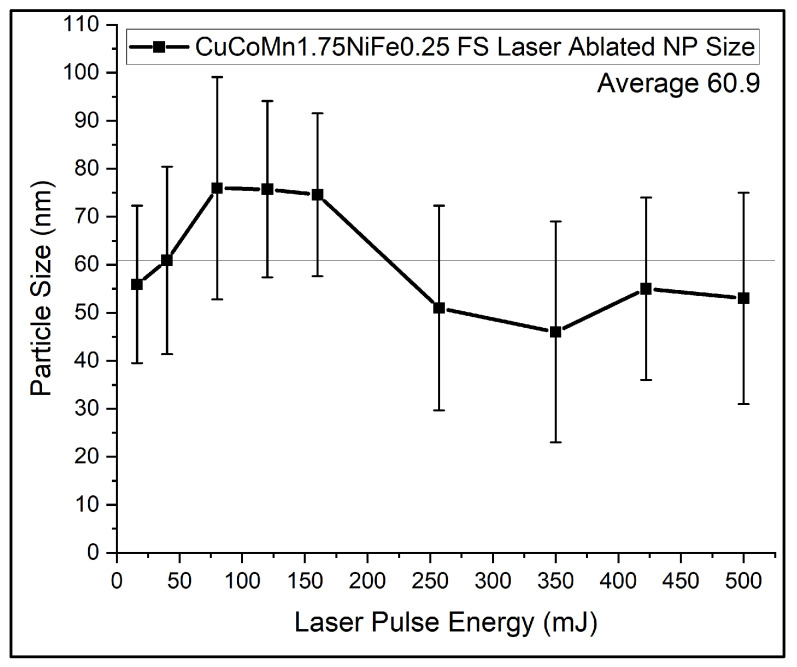
Size analysis of HEA nanoparticles.

Given that the nanoparticles exhibited a single-phase metal structure, ethanol was chosen as the preferred medium for ablation. The particle size analysis was initially conducted using SEM, with the results presented in Figure 5. Analysis of the SEM images reveals that while there is some variation in the size of the nanoparticles, they predominantly cluster within one standard deviation of the average size, approximately 60 nm. The slight decrease in mean size at higher laser fluences may be a result of laser-induced breakdown or an unusual welding phenomenon which will be discussed later in this document. For this statistic study shown in Figure 5, the chain particles were not calculated. For an optimized yield rate, the system’s production rate was tested at 5 W, which produced 10 mg per minute. Since the maximum power of the laser is 100 W, in principle, a linear scaling of 200 mg per minute rate can be feasible by multi-beam parallel processing. Therefore, the current method is scalable for a much higher production rate.

**Figure 6:**
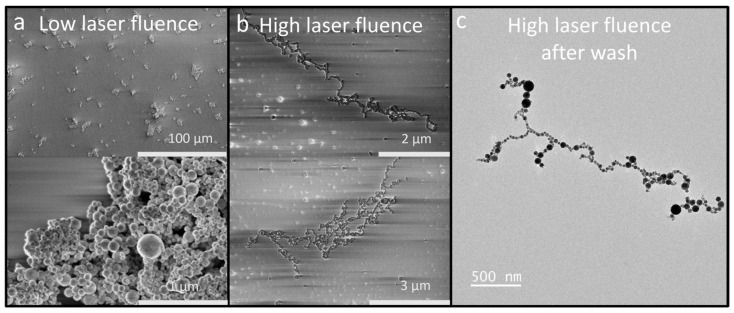
(**a**) SEM image of HEA nanoparticles at low laser power and fluence, (**b**) SEM image of HEA nanoparticle chains after ablation at high laser fluence, and (**c**) TEM image of high laser fluence chains after washing away organic material.

An unexpected phenomenon emerged during the size analysis, as illustrated in Figure 6. At pulse energies above 250 mJ, the nanoparticles exhibited a tendency to form elongated chains, extending up to microns in length. Figure 6a displays the common clustering of nanoparticles at lower laser fluences, aligning with typical expectations in nanoparticle analysis [52]. In contrast, Figure 6b reveals the formation of these extensive chains under higher laser powers. Initially, the hypothesis was that organic strands could be the cause of this clustering. This led to thorough and vigorous washing of the samples with deionized (DI) water to remove any potential organic residues. Despite this, subsequent TEM observations confirmed the persistence of the chaining phenomenon.

**Figure 7:**
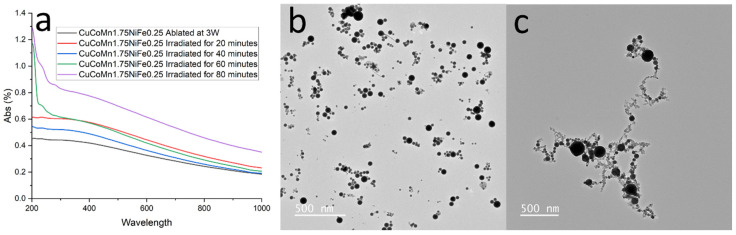
(**a**) UV-NIR spectra of nanoparticle solutions after fs irradiation. (**b**) TEM image of ethanol-ablated HEA nanoparticles. (**c**) TEM image of the same nanoparticle solution after fs irradiation.

To explore this phenomenon more thoroughly, an experiment was designed in which a laser was directed through a solution of nanoparticles while stirring continuously. Initially, the nanoparticles were ablated using low laser fluence, as depicted in Figure 6a and Figure 7b. This feature resulted in a random distribution of nanoparticles, a typical characteristic of nanoparticle agglomeration. Subsequently, the laser’s power was set to 50 W at 100 Hz, providing intense photon irradiation at a low density to the solution. The nanoparticles underwent irradiation at various time intervals, and after a period of 20 min, their characteristics were analyzed using UV-NIR spectroscopy, shown in Figure 7a. Following this process, TEM was employed to examine the particles synthesized in the solution, revealing the formation of nanoparticle chains akin to those observed in high fluence ablation, as demonstrated in Figure 7c. This experiment successfully confirmed that femtosecond laser irradiation can be utilized to promote the growth of nanoparticle chains. Such a process might offer advantageous properties, e.g., enhancing electrocatalytic effects by reducing resistance in the HEA catalyst. However, further research is required to establish these potential benefits conclusively. The notable high absorption observed in the UV region (~200 nm) of Figure 7a for the irradiated nanoparticle solutions requires further study to fully discern the underlying causes. Increased optical density at shorter wavelengths is indicative of light-scattering effects and plasma resonance absorption, both consistent with the formation of nanoparticle chains and aggregates. Additionally, residual organic compounds from ablation processing, as well as potential surface reconstruction of metal nanoparticles, can introduce electronic state transitions that absorb intensely at higher photon energies in the UV.

### 3.3. Laser Ablation and Joining Mechanisms

**Figure 8:**
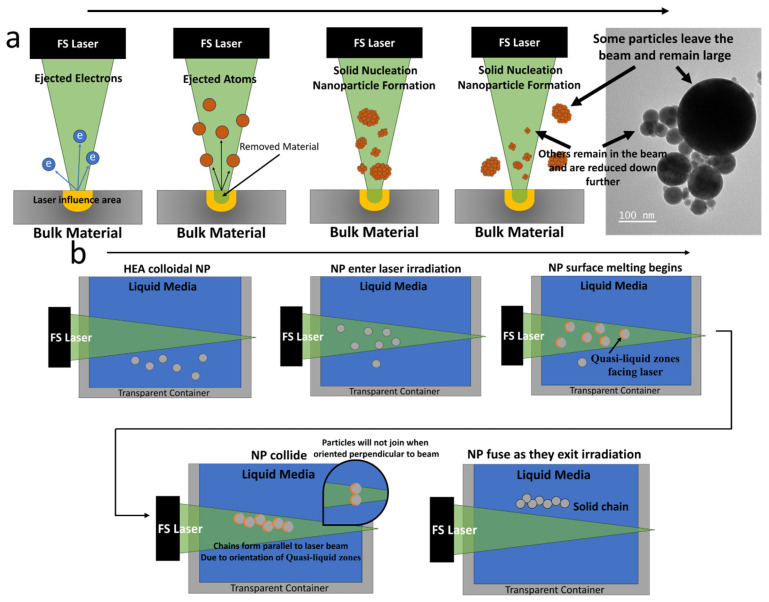
(**a**) Illustration of the laser ablation process for the formation of nanoparticles. (**b**) Depiction of surface nanojoining process.

**Figure 9:**
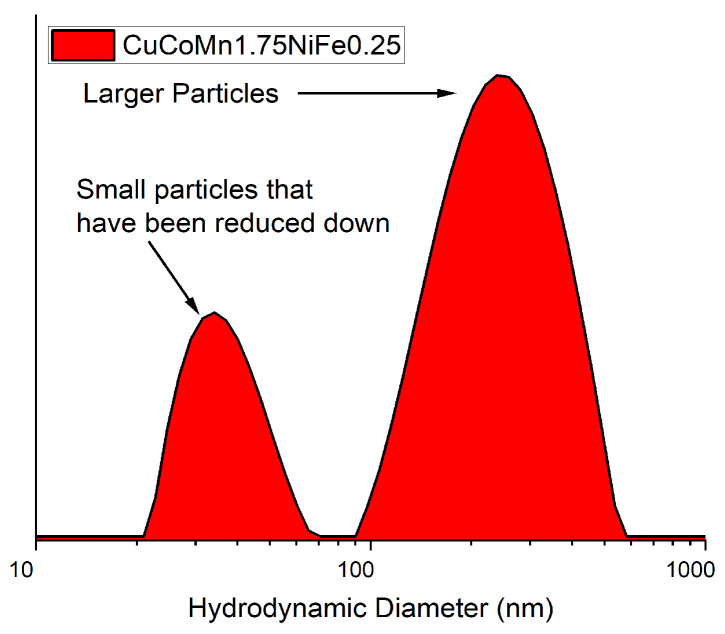
Hydrodynamic diameter of HEA nanoparticle solution.

Nonthermal ablation, pivotal in femtosecond laser-induced joining of nanoparticles, involves ultrafast interactions where femtosecond laser pulses primarily affect the material’s electrons. This rapid interaction leads to intense electron excitation and ionization, significantly impacting the material’s structure without notable lattice heating, while still increasing the energy [53,54]. This sudden energy boost causes some electrons to be ejected from the atom [36]. As a result [54], there is a significant imbalance between the positively charged protons and negatively charged electrons, leading to a Coulomb explosion. The Coulomb explosion refers to the phenomenon where positively charged ions repel each other due to the lack of sufficient electrons to balance the charge. This repulsion causes a rapid expansion and ejection of material from the surface, which is the key aspect of nonthermal ablation under femtosecond laser irradiation [55]. The process of electron ejection and subsequent Coulomb explosion, induced by femtosecond lasers, is crucial for understanding how these lasers modify a material’s structures at the nanoscale. Figure 8a depicts this laser ablation method followed by the formation of nanoparticles. The process unfolds in distinct stages. Initially, the target material absorbs energy from femtosecond laser pulses, leading to the excitation of electrons and the creation of a dense electron cloud near the surface. This electron cloud constitutes the initial stage of plasma formation, setting the stage for the subsequent Coulomb explosion. As the electron cloud gains energy, further ionization occurs, leading to the generation of plasma, a mixture of free electrons and ions. During the Coulomb explosion, the intense repulsive forces between the positively charged ions in the plasma lead to the rapid expansion and ejection of material from the target surface. This explosive release propels the atoms into the surrounding space. Following their expulsion into a liquid medium, these atoms undergo collision, cooling, slowing down, and nucleation, the process by which they combine and solidify, resulting in the formation of discrete solid particles. Notably, the fate of these particles varies; some remain within the laser beam, undergoing further fragmentation by the laser pulses and thereby reduction in size, while others exit the beam and maintain their larger dimensions. These are evident in the bimodal hydrodynamic diameter distribution scan using an Anton Paar Light Sizer, shown in Figure 9. Two maxima of the population are found at 40 nm and 270 nm. At higher repetition rates, increased laser pulses overlapping on trapped particles may enable secondary fragmentation, reducing sizes below the initial distribution. Factors such as repetition rate and target rotation speed alter pulse counts across ablation areas, likely influencing nanoparticle dimensions, composition uniformity, and microstructure through cumulative energy deposition effects.

On the other hand, nonthermal melting, a critical phase transition induced by low femtosecond laser fluence, plays a vital role in the joining of nanoparticles. The nonlinear adsorption induced by the femtosecond laser causes a shift in electron density, leading to a slight imbalance that does not reach the threshold of Coulomb explosion or electron ejection [36]. Instead, this imbalance also weakens the interatomic bonds, creating a quasi-liquid state at the irradiated spots. As a result, nanoparticles can merge or fuse at these quasi-liquid zones while maintaining their individual structural integrity and properties [36]. Significantly, this transition occurs without a corresponding rise in the lattice’s thermal energy, ensuring the nanoparticles’ structural integrity is preserved. Understanding this phenomenon can explain why our nanoparticles join when placed under relatively low laser fluence. The direct mechanism for our nanoparticle fusion is detailed in Figure 8b. In the process described, HEA nanoparticles are introduced into a zone of direct laser irradiation, where the density of the laser is intentionally kept low to avoid the aforementioned Coulomb explosions. Instead, the focus is on inducing surface melting of these nanoparticles. As the nanoparticles traverse this irradiated zone, the outermost layer undergoes a phase transformation into a quasi-liquid state due to the laser’s thermal influence. As the nanoparticles, now with molten surfaces, move within the laser field, they encounter and collide with each other. The surface liquid layers enable the nanoparticles to fuse with each other. Upon exiting the area of laser influence, the nanoparticles begin to cool down. This cooling results in the solidification of the quasi-liquid layers, effectively welding the nanoparticles together to form the long chains shown in Figure 6c and Figure 7c. These chains cannot de-weld due to surface melting not penetrating all the way through the fused zones. This fusion forms a consolidated mass. It is crucial to note the directional properties of this process. The formation of chains or aggregates of nanoparticles is highly dependent on the orientation of the liquid zones, their orientation to the beam, and the size of the laser beam. The orientation of these quasi-liquid zones is such that it is less possible to support perpendicular alignment relative to the laser beam. This directional limitation is further reinforced by the beam’s spatial constraints, which may restrict the chain length of joined particles. The light excitation of high-entropy nanoparticles may further shed light on the formation mechanism of fused particles. Also, the thermal accumulation effect due to high repetition may also contribute to laser-induced nanojoining [56,57].

### 3.4. Electrochemical Performance of HEA Nanoparticles

**Figure 10:**
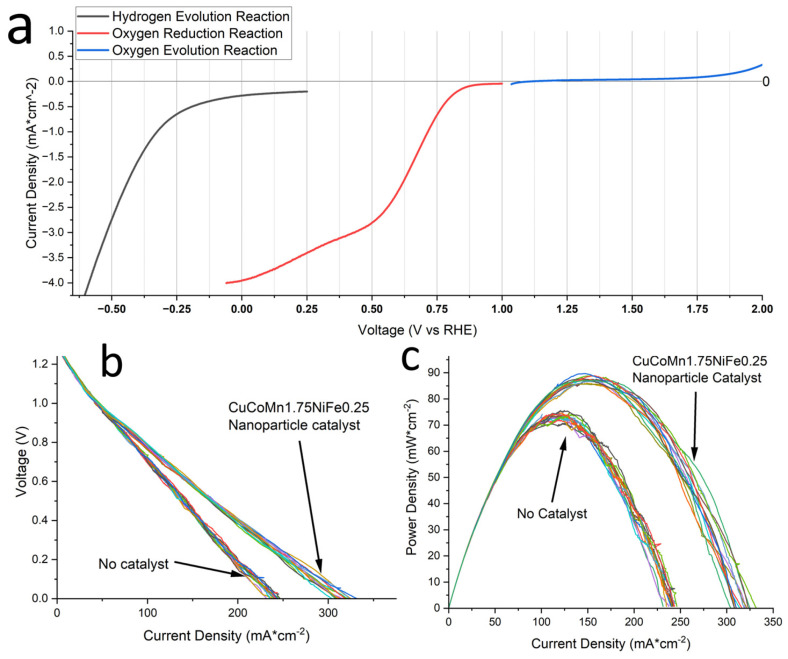
(**a**) LSV plots demonstrating multi-function catalysis of HEA nanoparticles, featuring hydrogen evolution, oxygen reduction, and oxygen evolution. (**b**) LSV curves for AAB showing output current density. (**c**) LSV curves for AAB showing output power density.

The exploration of HEA nanoparticles as electrocatalysts represents a burgeoning field of research with the potential to revolutionize energy conversion and storage technologies [2]. Figure 10a presents the LSV plots illustrating the multi-functional catalyst applications of the HEA nanoparticles. These curves reveal the particles’ ability to catalyze key reactions: HER, ORR, and OER. This multi-faceted catalytic capability opens new avenues for technological advancements. The multi-functional nature of these HEA nanoparticles as catalysts offers promising opportunities for their use in diverse energy technologies, including fuel cells, electrolyzers, and metal–air batteries. Their distinctive properties have the potential to enhance the efficiency, sustainability, and cost-effectiveness of energy conversion and storage systems.

The unique multi-elemental nature of HEAs contributes to a versatile electronic structure, which is beneficial for catalyzing oxygen reduction reactions in alkaline aluminum–air batteries [58]. This adaptability allows for the fine-tuning of electronic properties, which is crucial for optimizing the ORR process. The introduction of HEA nanoparticles as a catalyst in AABs offers promise to exploit these attributes for enhanced electrochemical performance. Building upon the framework of our investigation into aluminum–air batteries, we implemented these particles within our previously characterized batteries. This practical application aimed to evaluate the catalytic proficiency of synthesized nanoparticles. LSV yielded insightful data with a two-electrode configuration, as presented in Figure 10b, demonstrating a clear uptick in battery performance with the introduction of our nanoparticles. In this case, Al was the anode and HEA was deposited on carbon fiber paper as the cathode. Specifically, the LSV curves indicate a baseline performance of under 250 mA/cm^2^ without a catalyst (only KB and carbon Super-P). The integration of our HEA nanoparticles as the sole variable adjustment propelled the performance to approach 325 mA/cm^2^. A similar increase in the power density can be seen in Figure 10c. The maximum powder density increased from 70 mW/cm^2^ at a discharge current of 125 mA/cm^2^ to 90 mW/cm^2^ at a current density of 160 mA/cm^2^. This significant increase serves as a testament to the catalytic capabilities of these nanoparticles.

## 4. Conclusions

This study has established a refined method for producing high-purity high-entropy alloy nanoparticles through femtosecond laser ablation in liquid. Our approach has notably advanced the synthesis process of multi-component high-entropy alloying nanoparticles and achieved a significant production rate while maintaining nanoparticle integrity. The CuCoMn_1.75_NiFe_0.25_ nanoparticles developed here have showcased promising electrocatalytic properties, especially in enhancing the oxygen reduction reaction in alkaline aluminum–air batteries. The discovery of a unique nanoparticle joining mechanism through femtosecond laser irradiation has also emerged as a pivotal aspect of our research, illustrating the potential for controlled nanoparticle assembly and architecture for functional devices. This research work not only contributes to the fundamental understanding of HEA nanoparticles but also opens new avenues for the application of these materials in energy conversion technologies and beyond. Future investigations will aim to further optimize the electrocatalytic functionalities of these nanoparticles, potentially leading to breakthroughs in the efficiency and sustainability of energy systems. Ongoing efforts also seek to study the effects of pulse number on nanoparticle products through variation in laser parameters, including repetition rate and target rotation speed.

## Data Availability

In accordance with the MDPI research data policies, this article does not feature new data, nor are there datasets generated or analyzed during the study that could be shared publicly. The nature of this research did not involve the creation or use of any empirical data, experimental data, or datasets that could be archived in public repositories.

## Supplementary Materials

The following supporting information can be downloaded at: https://www.mdpi.com/article/10.3390/nano14060554/s1.
