# Supplementary material for: Synthesis and Unique Behaviors of High-Purity HEA Nanoparticles Using Femtosecond Laser Ablation
Source: Nanomaterials (Basel). 2024 Mar 21;14(6):554. doi: 10.3390/nano14060554 (PMC10974168; doi:10.3390/nano14060554)
Supplement: Supplementary file 1 [file nanomaterials-14-00554-s001.zip › nanomaterials-2880746-supplementary.pdf]

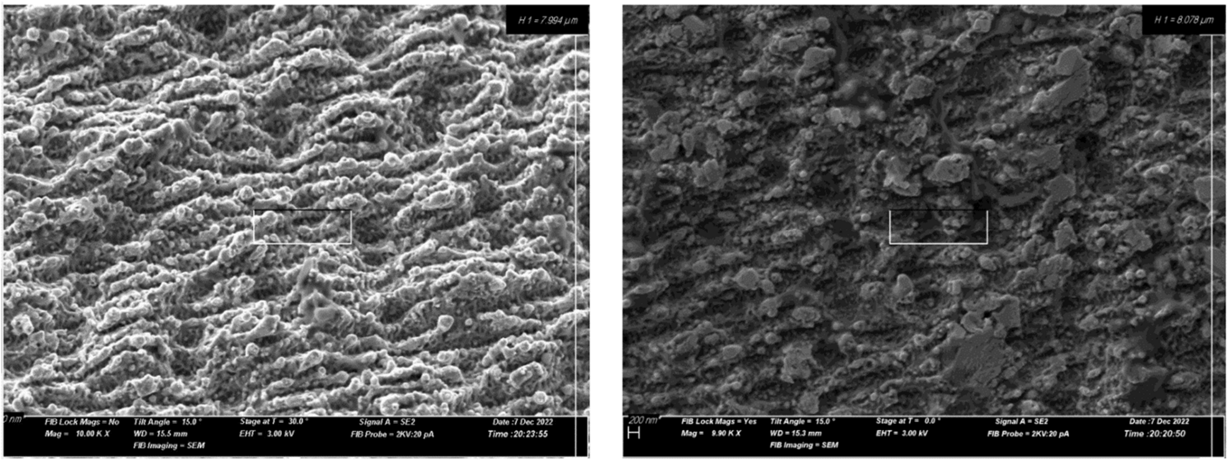

Figure S1 Morphology of ablated surface.

Figure S1 presents Laser-induced periodic surface ripples formed through femtosecond ablation on HEA target surface. Periodicity of ~200 nm observed, in line with laser wavelength expected to induce such self-organized structures.
